# CORRECTION

**DOI:** 10.1111/cas.15343

**Published:** 2022-05-24

In an article^1^ titled “MicroRNA‐16 inhibits glioma cell growth and invasion through suppression of BCL2 and the nuclear factor‐κB1/MMP9 signaling pathway” by Tian‐Quan Yang, Xiao‐Jun Lu, Ting‐Feng Wu, Da‐Dong Ding, Zhao‐Hui Zhao, Gui‐Lin Chen, Xue‐Shun Xie, Bin Li, Yong‐Xin Wei, Ling‐Chuan Guo, Yu Zhang, Yu‐Lun Huang, You‐Xin Zhou, Zi‐Wei Du, Figure 4a was published with incorrect images. The correct figure is presented below.

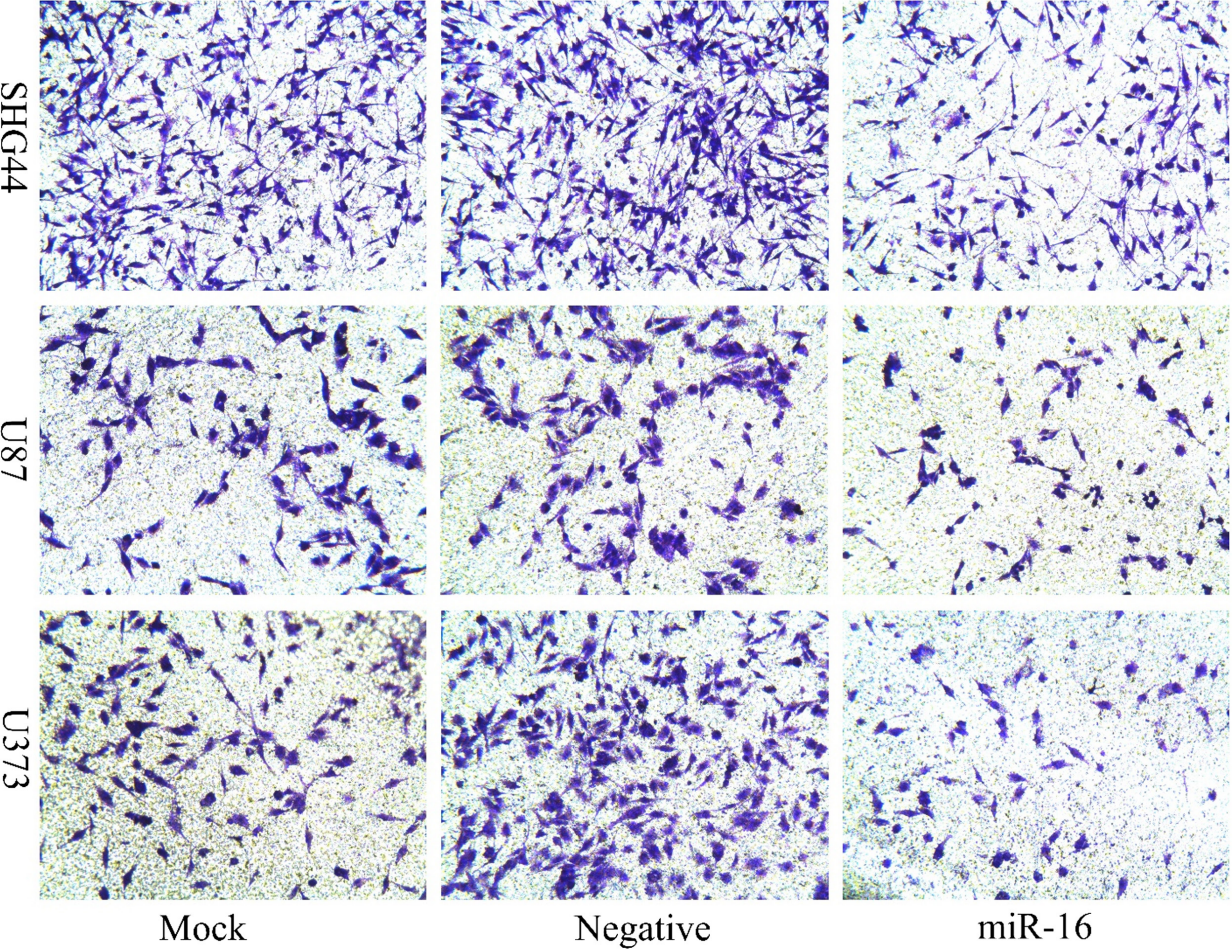

The authors apologize for the error.
